# Loss of *Nicotinamide nucleotide transhydrogenase* sensitizes embryos to ethanol-induced neural crest and neural apoptosis via generation of reactive oxygen species

**DOI:** 10.3389/fnins.2023.1154621

**Published:** 2023-06-09

**Authors:** Rayna Mazumdar, Johann K. Eberhart

**Affiliations:** ^1^Department of Molecular Biosciences, School of Natural Sciences, University of Texas at Austin, Austin, TX, United States; ^2^Waggoner Center for Alcohol and Addiction Research, School of Pharmacy, University of Texas at Austin, Austin, TX, United States

**Keywords:** FASD, neural crest cells, oxidative stress, *nnt*, ethanol, brain, gene–environment interactions

## Abstract

Fetal alcohol spectrum disorders (FASD) are a continuum of birth defects caused by prenatal alcohol exposure. FASD are the most common environmentally induced birth defect and are highly variable. The genetics of an individual influence the severity of their FASD phenotype. However, the genes that sensitize an individual to ethanol-induced birth defects are largely unknown. The ethanol-sensitive mouse substrain, C57/B6J, carries several known mutations including one in *Nicotinamide nucleotide transhydrogenase* (*Nnt*). Nnt is a mitochondrial transhydrogenase thought to have an important role in detoxifying reactive oxygen species (ROS) and ROS has been implicated in ethanol teratogenesis. To directly test the role of Nnt in ethanol teratogenesis, we generated zebrafish *nnt* mutants via CRISPR/Cas9. Zebrafish embryos were dosed with varying concentrations of ethanol across different timepoints and assessed for craniofacial malformations. We utilized a ROS assay to determine if this could be a contributing factor of these malformations. We found that exposed and unexposed mutants had higher levels of ROS compared to their wildtype counterparts. When treated with ethanol, *nnt* mutants experienced elevated apoptosis in the brain and neural crest, a defect that was rescued by administration of the antioxidant, N-acetyl cysteine (NAC). NAC treatment also rescued most craniofacial malformations. Altogether this research demonstrates that ethanol-induced oxidative stress leads to craniofacial and neural defects due to apoptosis in *nnt* mutants. This research further supports the growing body of evidence implicating oxidative stress in ethanol teratogenesis. These findings suggest that antioxidants can be used as a potential therapeutic in the treatment of FASD.

## Introduction

Fetal alcohol spectrum disorder (FASD) encompasses the full range of birth defects caused by prenatal alcohol exposure. FASD is thought to be the most widespread of environmentally-induced birth defects in humans, affecting between 2 and 5% of all live births in the U.S (May et al., 2014a). While alcohol exposure is necessary to induce this spectrum of deleterious effects, the outcomes of these exposures are highly variable. FASD can range in severity from neurodevelopmental deficits and behavioral abnormalities as seen in Alcohol Related Neurodevelopmental Disorder, to craniofacial defects such as microphthalmia, microcephaly, smoothened philtrum, and hypoplastic mandible typical of Fetal Alcohol Syndrome (Stratton et al., 1996).

While exposure to alcohol is necessary to give rise to these developmental defects, genetics modulate the sensitivity to ethanol. Of pregnancies with known heavy alcohol exposure, only 4.3% of children develop Fetal Alcohol Syndrome (Abel, 1995). Twin studies have provided further evidence for the role of genetics in modulating the outcomes of alcohol exposures. Studies of monozygotic and dizygotic twins have shown that monozygotic twins were 100% concordant in their FAS diagnosis, while dizygotic twins were only 56–64% concordant despite experiencing the same prenatal environment (Streissguth and Dehaene, 1993; Astley Hemingway et al., 2018). We continue to make strides toward understanding the genetic influence on ethanol teratogenesis, but our comprehension remains extremely limited.

Strain differences within animal model systems provided some of the early evidence for the importance of genetics in ethanol-induced birth defects. C57BL/6J mice are sensitized to ethanol-induced teratogenesis compared to their C57BL/6N counterparts. Among other substrain differences, C57BL/6J mice have a 5-exon deletion in *Nicotinamide nucleotide transhydrogenase* (*Nnt*) (Freeman et al., 2006). Nnt is a transhydrogenase in the inner mitochondrial membrane that is important in ATP synthesis and the reduction of NADP+ to NADPH (Hoek and Rydström, 1988). C57BL/6J experience with the *Nnt* mutation have a nondetectable level of the protein and impaired glucose clearance and H_2_O_2_ metabolism (Huang et al., 2006; Ronchi et al., 2013).

Nnt can sequester reactive oxygen species (ROS) via NADPH-mediated reduction of the oxidized form of glutathione (Rush et al., 1985). Failure to sequester ROS can lead to oxidative stress. Oxidative stress can induce apoptosis in cells through the increased production of free radicals, peroxidation of membranes, and mitochondrial dysfunction (Zamzami et al., 1995). The metabolism of ethanol by alcohol dehydrogenase (ADH) creates acetaldehyde, which itself can lead to overproduction of ROS (Chu et al., 2007; Yan and Zhao, 2020) Reduction of NNT activity leads to lower NADPH concentration and lower oxidized glutathione/reduced glutathione conversion (Sheeran et al., 2010). When *NNT* is knocked down in cell culture, proliferation is decreased and the NADH/NAD+ ratio is increased relative to the control, likely because this oxidation step is coupled with the reduction of NADP+ to NADPH (Ho et al., 2017). Thus, loss of *Nnt* may sensitize embryos to ethanol teratogenesis.

While *NNT* dysfunction has been implicated in human diseases such as glucocorticoid deficiency 1, there has yet to be a direct exploration of its interaction with ethanol during development (Meimaridou et al., 2012). Zebrafish provide an excellent system to study ethanol teratogenesis due to external fertilization, ease of determining developmental age, large clutch sizes, rapid development, availability of transgenic approaches, and a high level of conservation to the human genome. Thus, the zebrafish serves as an exceptional model to explore the effects and mechanisms of ethanol-induced birth defects.

Here, we demonstrate that loss of *nnt* sensitizes embryos to ethanol-induced teratogenesis. Ethanol induces a range of craniofacial phenotypes ranging in severity across ethanol concentration, dosing windows, and genotype, with ethanol-exposed *nnt* mutants having the most severe defects in the facial skeleton. Mutants appear most sensitive to ethanol from 6 to 24 h post-fertilization (hpf). Apoptosis was significantly elevated in the neural crest of control and ethanol-treated *nnt* mutants compared to their wildtype siblings, although proliferation was not significantly changed across genotype or treatment. Apoptosis was also significantly increased in the brains of ethanol-exposed *nnt* mutants. Levels of ROS predictably increased across treatment and genotype, with ethanol-exposed mutants having the highest levels. Apoptosis and craniofacial abnormalities were largely rescued by concurrent antioxidant administration using N-acetyl cysteine (NAC). Our findings demonstrate that *nnt* modifies susceptibility to embryonic ethanol exposure, providing more evidence for the role of oxidative stress in ethanol teratogenesis.

## Materials and methods

### Zebrafish husbandry

All animal research was performed in accordance with a protocol approved by the University of Texas at Austin Institutional Animal Care and Use Committee. Embryos were incubated at 28°C in embryo media (Westerfield, 1993). Zebrafish were staged according to the zebrafish developmental staging series (Kimmel et al., 1995). All zebrafish stocks used were derived from the AB wild-type genetic background.

### CRISPR-Cas9

We utilized ZiFiT Targeter^1^ to identify gRNA binding sites for *nnt*. We made *nnt* gRNA via MEGAscript T7 Kit (Invitrogen) using a described protocol (Jao et al., 2013). The oligo used to generate the gRNA is aattaatacgactcactataGGCCTCATGAACTCCTAGAGgtttta gagctagaaatagc (capitalized nucleotides code for the gRNA). We injected embryos with a 2 nl bolus of a cocktail containing: 500 ng/μl *Cas9* protein (IDT) and 250 ng/μl *nnt* gRNA in water with 0.2% phenol red.

One F1 individual, with a 74 base pair deletion in *nnt* (designated *nnt^au111^* and we refer to the allele as *nnt^−^* for clarity), was backcrossed to AB to produce a stable line. To assess whether *nnt* expression was absent in the mutant embryos, fluorescent *in situ* hybridization was performed using a Molecular Instruments probe on 24 hpf zebrafish embryos according to manufacturer’s protocol (Choi et al., 2018).

### Chemical treatments

Embryos were treated with ethanol at various timepoints from 6 to 24 hpf, 24–48 hpf, 48–72 hpf, and 6 hpf-5 days post-fertilization (dpf), as well as with different ethanol concentrations in embryo medium (0.5, 0.75, 1, 1.25, and 1.5% ethanol). Embryos were also treated with 1 mM NAC (Sigma-Aldrich) and 1% ethanol concurrently from 6 to 24 hpf. Previous studies have shown that tissue levels are 1/4–1/3 of the dose of ethanol, meaning a dose of 1% ethanol is approximately equivalent to a blood alcohol concentration of 0.18 to 0.21 (Lovely et al., 2014). NAC has been used in animal models and the 1 mM dosage is based on previous literature (Ni et al., 2020).

### Skeletal analyses

Zebrafish were stained at 5 dpf with Alcian Blue and Alizarian Red (Walker and Kimmel, 2007). Whole and flat-mount craniofacial images were then taken on the Zeiss AxioImager.A1 compound microscope with a Zeiss AxioCam HRc camera. Whole body images were taken on an Olympus SZX7 microscope with an Olympus DP22 camera.

### TUNEL staining

Cell death was assessed using the Biotium *CF*® 640R TUNEL Assay Apoptosis Detection Kit. Following chemical treatment, embryos were fixed at 36 hpf in 4% paraformaldehyde (PFA) overnight at 4°C with gentle agitation. Embryos were dehydrated and rehydrated with serial dilutions of methanol (MeOH) and Phosphate Buffered Saline with 0.5% Triton-X (PBTx) at room temperature. Embryos were permeabilized with a 25 μg/ml solution of proteinase K for 5 min and fixed with 4% PFA for 20 min. After washing with PBTx, embryos were incubated with the TUNEL solution (5 μl of enzyme in 45 μl buffer) for 3 h at 37°C with gentle agitation. Embryos were washed with PBTx and postfixed with 4% PFA for 30 min. Embryos were washed with PBTx and stored in 1X Phosphate buffered saline (PBS) for imaging.

### Phospho-histone H3 (pHH3) staining

Cell proliferation was assessed using the pHH3 (Ser10) (D2C8) XP^®^ Rabbit mAb (Cell Signaling Technology). Embryos were fixed at 36 hpf in 4% PFA overnight at 4°C with gentle agitation. Embryos were dehydrated and rehydrated with serial dilutions of MeOH and PBTx at room temperature. Embryos were washed 4 times for 30 min each in IB buffer (1% BSA, 0.5% TritonX100, and 1% DMSO in 1X PBS). Embryos were incubated with IB containing 5% Normal goat serum (NGS) for 30 min. Embryos were then incubated with the primary antibody in IB + 5% NGS at a 1:250 dilution overnight at 4°C with gentle agitation. Embryos were washed with IB once for 30 min, then with IB +5% NGS for 30 min. Embryos were incubated with the Invitrogen Goat anti-Rabbit IgG (H + L) Secondary Antibody, Alexa Fluor™ 568 in IB + 5% NGS at a 1:500 dilution overnight at 4°C with gentle agitation. Embryos were washed 3 times with PBTx for 15 min each and postfixed in 4% PFA for 30 min. Embryos were washed 3 times with 1X PBS for 5 min each and stored in 1X PBS for imaging.

### ROS assay

ROS concentration was assessed in embryos using the CellROX® ROS Assay kit (Thermo Scientific). 24 hpf embryos were incubated with a 2.5 μM enzyme solution for 30 min at 28°C, while 48 hpf embryos were incubated with a 5 μM enzyme solution for 15 min at 28°C. Embryos were washed with 1X PBS and imaged.

### Image analysis

Fluorescent embryos were imaged on a Zeiss LSM 710 Confocal. ImageJ was used to quantify apoptotic and proliferating cells, ROS fluorescence, and craniofacial and body length measurements.

### Statistical analysis

A two-tailed Fisher’s exact test of independence was used to determine if the occurrence of craniofacial defects between genotypes was statistically significant. Two-way ANOVA with Tukey’s multiple comparisons correction was used for analysis of the ROS assay, TUNEL data, craniofacial measurements, proliferation, and body length. All graphs and statistical analyses were run using Graphpad Prism 9.

## Results

### Zebrafish *nnt* mutants are sensitized to ethanol teratogenesis

**Figure 1:**
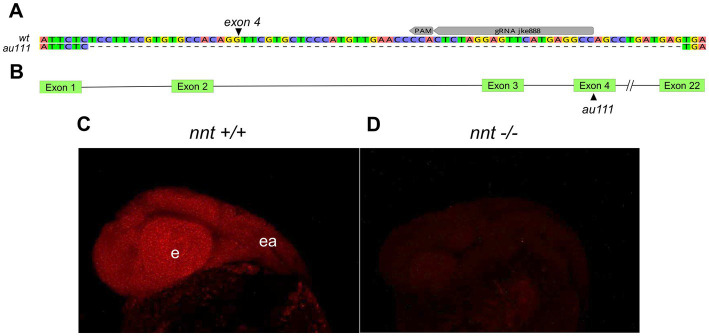
Generation of a zebrafish *nnt mutation*. **(A)** Schematic depicting the 74 bp deletion in exon 4 of *nnt*. The gRNA binding site (gRNA jke888) and PAM sequence are noted. **(B)** Location of the mutation (designated *au111*) relative to the 22 exon long gene. **(C)** Confocal images of fluorescent *in situ* hybridization staining for *nnt-*specific probe in 24 hpf wildtype and **(D)** mutant embryos, demonstrating a lack of transcript in *nnt* mutants, suggesting extensive non-sense mediated decay of the mutant transcript. Anterior is left, dorsal is up, e: eye, ea: ear.

To generate zebrafish *nnt* mutants, we injected CRISPR/Cas9 reagents into 1 cell-stage embryos and raised them to adulthood. The gRNA targeted exon 4, out of 22 (Figures 1A,B). One F0 fish gave rise to progeny harboring a 74 base pair deletion (Figure 1A) resulting in a frame shift and premature termination sequence.

Such an early stop codon would be anticipated to trigger nonsense-mediated decay of the mutant transcript. To determine if there was a loss of *nnt* mRNA, fluorescent *in situ* hybridization was performed on 24 hpf embryos. All wildtype and heterozygote embryos displayed bright and ubiquitous expression consistent with previous data (Thisse and Thisse, 2004). However, no transcript was detected in the *nnt* mutant, confirming that *nnt* expression is absent in the mutant embryos (Figures 1C,D).

**Figure 2:**
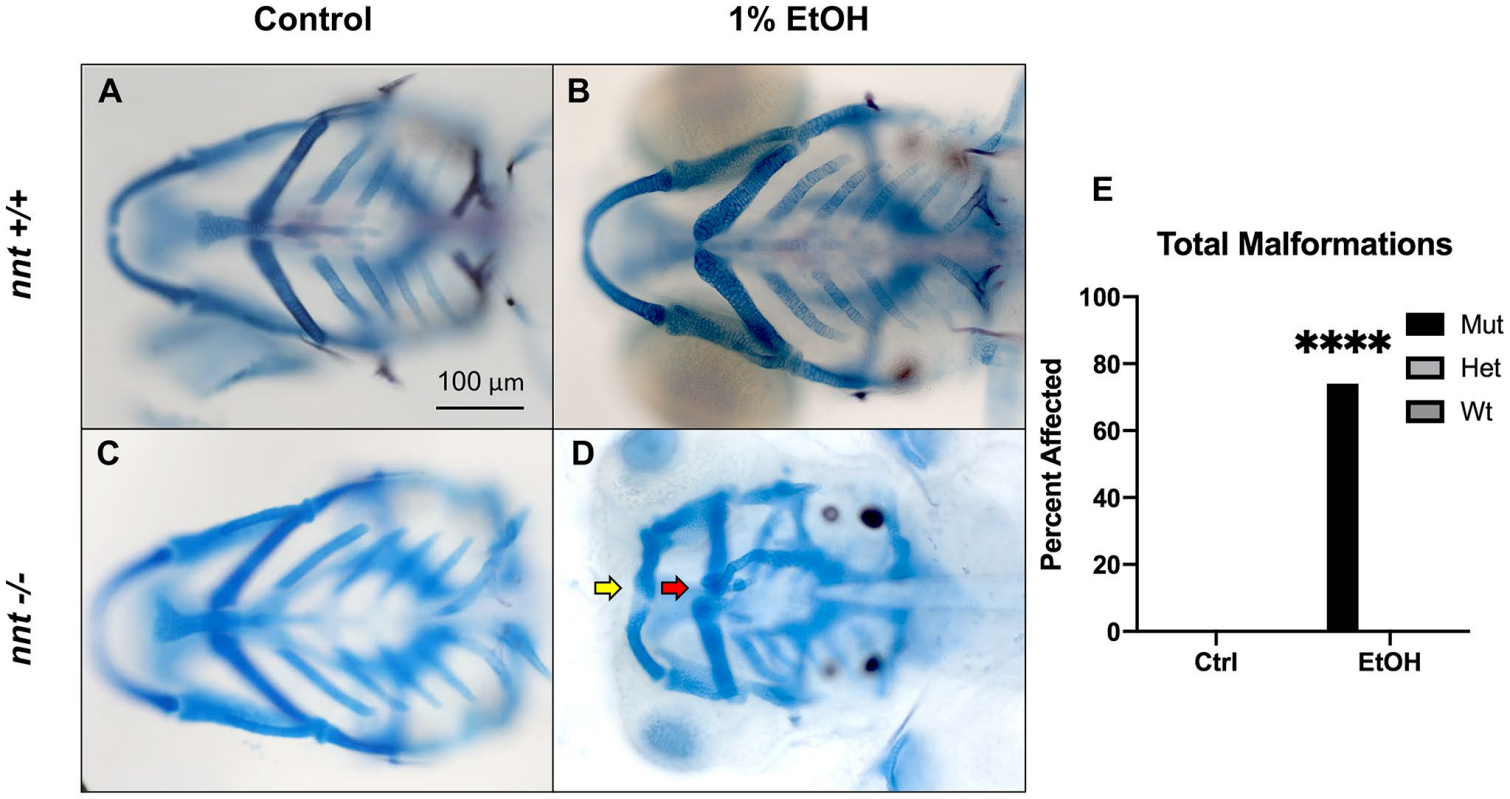
Loss of *nnt* sensitizes embryos to ethanol teratogenesis. **(A–D)** Alcian Blue/Alizarian Red-stained zebrafish at 5 dpf treated from 6 hpf- 5 dpf. All images are ventral views with anterior to the left. **(A,B)** Control and ethanol-treated wildtypes and **(C,D)** *nnt* mutants. Both the untreated and 1% ethanol dosed wt embryos appear phenotypically normal. **(C)** Untreated *nnt* mutant with a typical phenotype akin to wt. **(D)** Ethanol-exposed *nnt* mutant zebrafish with aberrant craniofacial phenotype consisting of a hypoplastic Meckel’s cartilage (yellow arrow), deformed ceratohyal (red arrow), microphthalmia, and microcephaly. **(E)** Quantification of the percentage of fish with craniofacial defects in each group (Fischer’s exact test, n ≥ 20 per group, ****p < .0001). Only ethanol-treated mutants displayed craniofacial defects, a statistically significant increase. EtOH: ethanol, Ctrl: control, mut: mutant, het: heterozygote, Wt: wildtype.

To assess whether *nnt* mutation predisposes embryos to ethanol-induced defects, embryos were dosed with 1% ethanol from 6 hpf-5 dpf (Figures 2A,D). As we have shown previously, ethanol-exposed wild-type fish are not discernable from unexposed wild-types (Figures 2A,B, *n* = 22 and *n* = 29, respectively). Similarly, unexposed *nnt* mutants are indistinguishable from wild-types (Figure 2C, *n* = 24). Indeed, unexposed *nnt* mutants are viable and fertile. In contrast, 74% (17/23) of ethanol-exposed mutant zebrafish had profound craniofacial defects (Figure 2D). This is a significant increase compared to wildtypes (*p* < 0.0001) (Figure 2E). These defects include reduced jaw (Meckel’s cartilage) and ceratohyal (Figure 2D). Ethanol-exposed mutants also have smaller eyes and heads as quantified in Supplementary 1 respectively. Ethanol-exposed mutants had significantly smaller eyes (*p* < 0.0001), consistent with data showing ethanol-exposed C57/B6J mice have smaller eyes compared to untreated embryos (Parnell et al., 2010). Ethanol-exposed fish survived to 5 dpf, indicating that there was not elevated embryonic mortality in the mutants. This demonstrates that *nnt* mutants are more susceptible to ethanol teratogenesis.

### The effects of the of *nnt* mutation on ethanol teratogenicity is dependent on time and dosage

**Figure 3:**
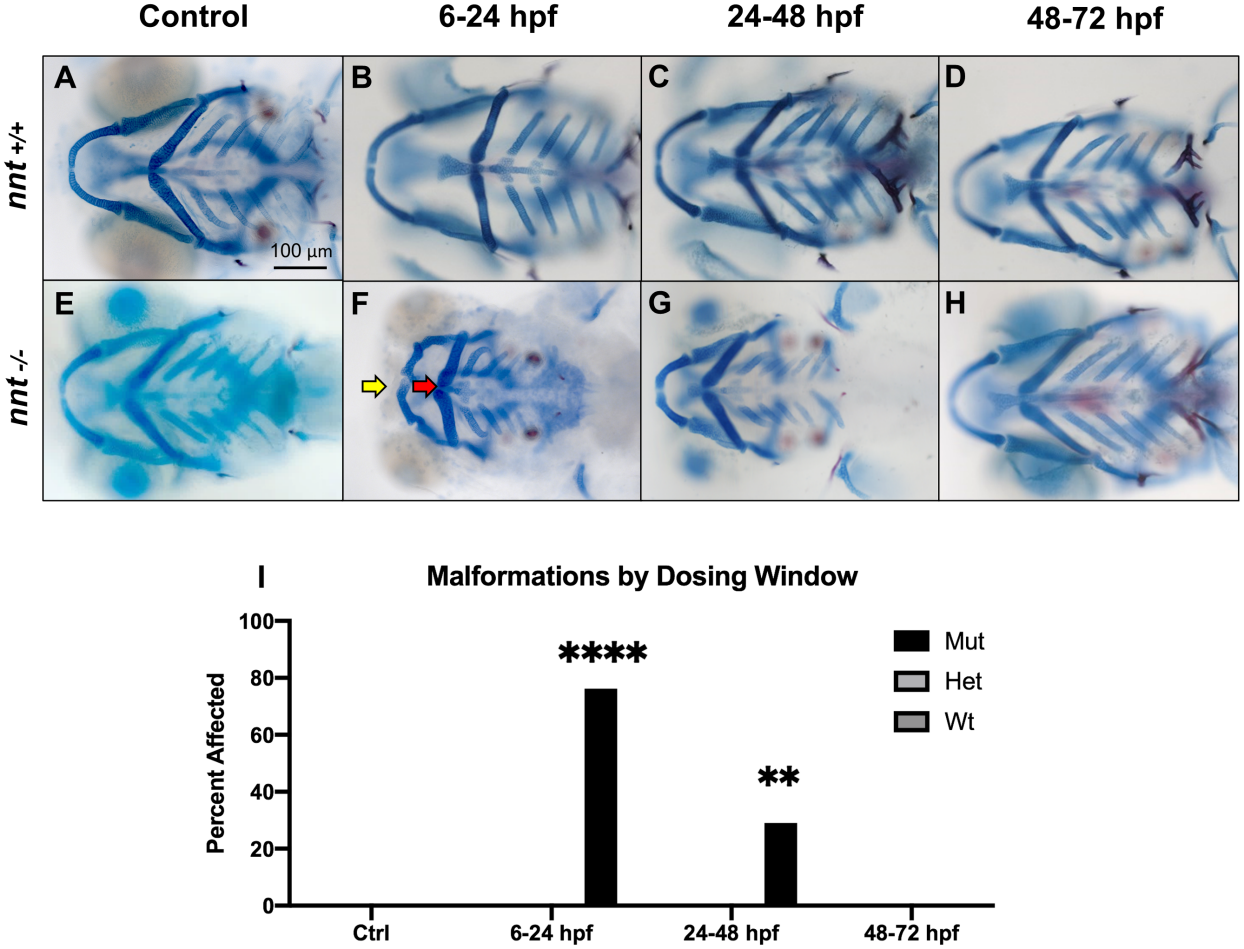
*nnt* mutants appear sensitized to ethanol teratogenesis from 6 to 24 hpf. Embryos were treated with 1% ethanol at different timepoints **(A–H)** to establish a critical period of ethanol teratogenesis. All images are ventral views with anterior to the left. Control (unexposed) wildtype and mutant **(A, E)** appear phenotypically normal. Wildtype embryos dosed across all timepoints **(B–D)** appear phenotypically normal. **(F)** Mutant embryos dosed at 6–24 hpf had stark craniofacial defects consisting of a hypoplastic Meckel’s cartilage (yellow arrow), deformed ceratohyal (red arrow), microphthalmia, and microcephaly. **(G)** Mutant embryos dosed at 24–48 hpf had no apparent defects in craniofacial morphology, but did have microcephaly and reductions in the size of skeletal elements. **(H)** Mutant embryos dosed at 48–72 hpf had no apparent defects. **(I)** Quantification of the percentage of fish with craniofacial defects in each group. (Fischer’s exact test, *n* ≥ 20 per group, **p  < 0.01, ****p  < .0001). The most highly statistical difference was observed at 6–24 hpf. Mut: mutant, Het: heterozygote, Wt: wildtype.

To identify the critical period of exposure, zebrafish embryos were dosed with 1% ethanol at different timepoints (Figures 3A–H). Of the mutants exposed from 6 to 24 hpf, 76% (16/21) had craniofacial abnormalities similar to those observed within the longer treatment window, a significant increase from the respective wildtype (*p* < 0.0001) (Figures 3F,I). In the larvae treated from 24 to 48 hpf, there was not a gross craniofacial phenotype, but 29% (9/31) had cardiac edema and smaller than average faces (Figure 3G). The incidence of these phenotypes was significantly higher in the mutants relative to exposed wildtype (*p* = 0.0020) (Figure 3I). Finally, in the 48–72 hpf treatment group, all 29 embryos appeared craniofacially typical and were otherwise indistinguishable from the wildtype (Figure 3H). Thus, the critical period of ethanol exposure for *nnt* mutants is between gastrulation and the onset of the pharyngula period.

**Figure 4:**
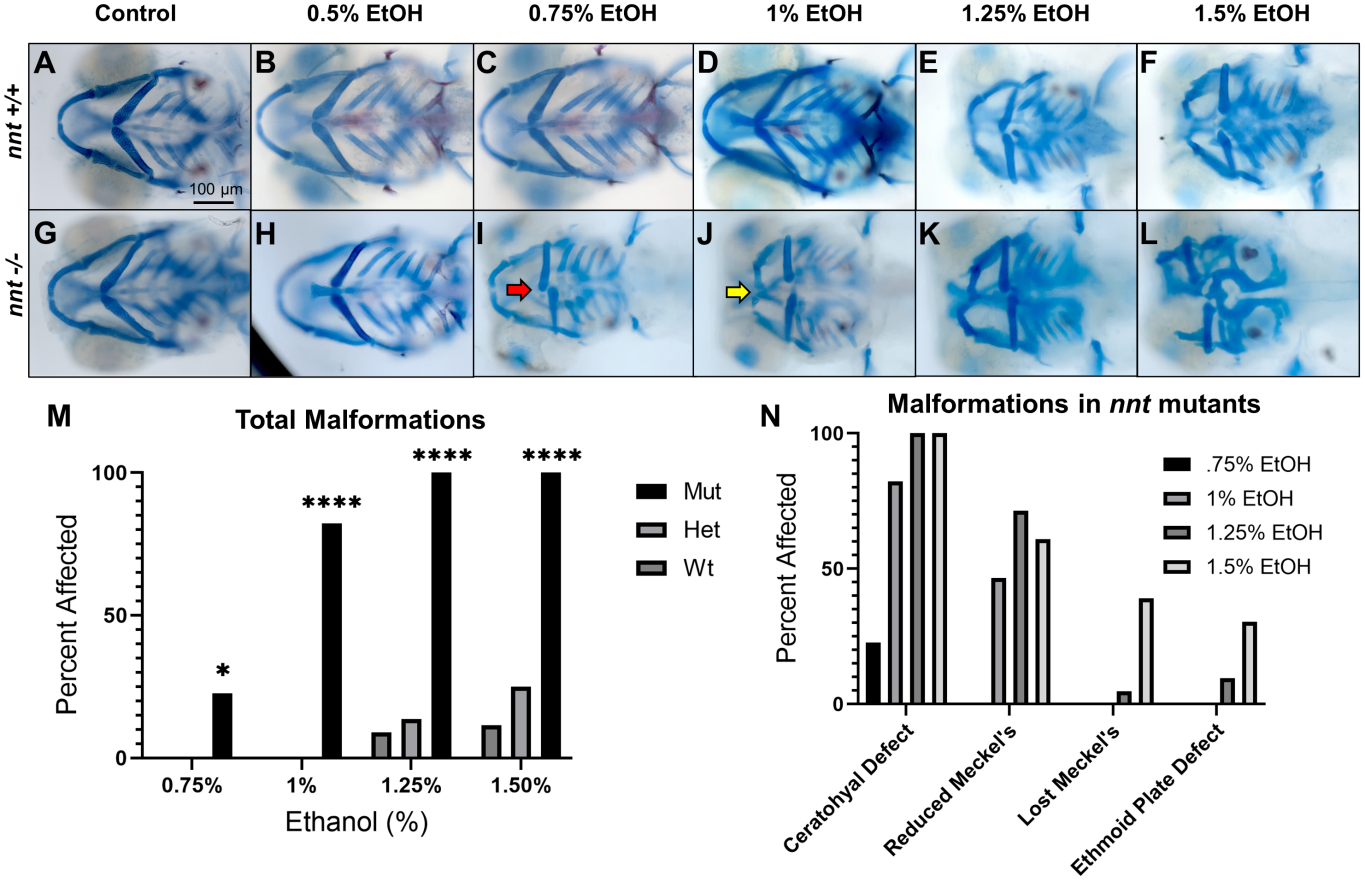
Severity of *nnt* malformations increases with ethanol concentration. **(A–L)** Untreated and ethanol-exposed Alcian Blue/ Alizarian Red-stained zebrafish embryos at 5 dpf treated from 6 hpf- 24 hpf. All images are ventral views with anterior to the left. **(A,B,G,H)** Wildtypes and mutants appear phenotypically normal at concentrations of 0 and 0.5% ethanol. **(C,I)** At 0.75% ethanol, wildtypes still appear normal, but 22.7% of mutants have ceratohyal cartilages that do not extend anteriorly (red arrow). **(D,J)** At 1% ethanol, the wildtypes remain normal, but 82.2% of nnt mutants had gross craniofacial defects, including a hypoplastic Meckel’s cartilage (yellow arrow). **(E,F,K,L)**.. At 1.25 and 1.5% craniofacial defects become apparent in the wildtypes, and there is increased severity of the craniofacial defects in the mutants. **(M)** A bar chart depicting total craniofacial malformations across genotype and ethanol concentration (Fisher’s exact test, *n* ≥ 20 per group, *p  < 0.05, ****p  < 0.0001). **(N)** A bar chart depicting the types of craniofacial malformations seen in mutants at different ethanol concentrations. EtOH: ethanol, Mut: mutant, Het: heterozygote, Wt: wildtype.

To further characterize and quantify the sensitivity of *nnt* mutants to ethanol, embryos were dosed with a range of ethanol concentrations. Embryos from heterozygous crosses were treated with 0, 0.5, 0.75, 1, 1.25%, or 1.5% ethanol solutions from 6 to 24 hpf (Figures 4A–L). In wildtype and heterozygous fish, there were no phenotypic defects observed at doses below 1.25% ethanol. At 1.25% ethanol, 8.70% (2/23) of wildtype and 13.6% (6/44) of heterozygous fish had craniofacial defects (Figures 4E,K,M). These percentages rose to 11.54% (3/26) and 25% (10/40), at 1.5% ethanol (Figures 4F,L,M). Thus, consistent with previous studies, 1% ethanol does not cause gross craniofacial defects in wildtypes, and it appears that *nnt* heterozygosity does not significantly sensitize embryos to this dose of ethanol (McCarthy et al., 2013; Swartz et al., 2014; Everson et al., 2020; Fish et al., 2021).

There was a range of phenotypes across most concentrations of ethanol in *nnt* mutants. While there were no craniofacial defects at 0.5% ethanol, at 0.75% ethanol, there was a significantly higher amount of craniofacial defects in the mutants compared to the 0.75% ethanol treated wildtypes (*p* = 0.0219) (Figures 4C,I,M). 21.74% (5/23) of treated *nnt* mutant larvae had craniofacial malformations. In all of these fish the malformation was the same: the ceratohyal cartilages fail to meet appropriately at the midline and do not angle anteriorly. This suggests that the ceratohyal is the most easily perturbed skeletal element in *nnt* mutants (Figure 4N).

At 1% ethanol, craniofacial malformations in *nnt* mutants increased substantially in penetrance and severity, with total malformations being significantly higher in the mutant compared to the wildtype (*p* < 0.0001) (Figures 4D,J,M). The ceratohyal defect, described above, was present in all fish with malformations 82.22% (37/45). Additionally, a subset of these fish, 46.67% (21/45), also had a reduced Meckel’s cartilage (Figure 4N). The reduced Meckel’s phenotype consisted of a Meckel’s cartilage that was shorter and wider than the typical anteriorly elongated structure in wildtype.

In the mutants treated with 1.25% ethanol, 100% (21/21) treated embryos had craniofacial defects, a significant amount compared to their wildtype counterparts (*p* < 0.0001) (Figures 4E,K,M). Again, the ceratohyal defect was the most common 100% (21/21). 71.4% (15/21) also had a reduced Meckel’s and 4.8% (1/21) having a loss of Meckel’s on one side of the face. At this dose, phenotypes were expanded to the neurocranium with 9.5% (2/21) having ethmoid plate defects (Figure 4N). These ethmoid plate defects were characterized by a small, pointed ethmoid plate replacing the typically wide and triangular ethmoid plate seen in wildtypes. Of the 2 embryos with ethmoid plate defects, one had a loss of Meckel’s and the other had a reduced Meckel’s. At lower doses there were no discernable defects in the wildtype, however at 1.25% ethanol, defects were seen in the wildtypes as well, with 8.70% (2/23) embryos displaying ceratohyal and reduced Meckel’s phenotype.

Finally, in the mutant group that was treated with 1.5% ethanol from 6 to 24 hpf, there was an even wider range of phenotypes, with the total malformations in mutants remaining significantly higher than the wildtype (*p* < 0.0001) (Figures 4F,L,M). 100% (23/23) of treated mutants had ceratohyal defects. All fish also had defects to Meckel’s cartilage, with 60.9% (14/23) having reduced Meckel’s and 39.1% (9/23) having lost Meckel’s. The number of fish with the ethmoid plate defect also rose, to 30.4% (7/23) (Figure 4N). Wildtypes at this dose were also disrupted, as 11.54% (3/26) embryos had the ceratohyal and reduced Meckel’s phenotype.

### ROS elevation underlies ethanol-induced defects in *nnt* mutants

We hypothesize that *nnt* mutants are more susceptible to the deleterious effects of ethanol exposure due to an inability to tolerate increased oxidative stress. Therefore, we quantified the levels of ROS and apoptosis and we tested the effects of antioxidant (NAC) administration on *nnt* mutant phenotypes.

NAC is a powerful antioxidant and, together with glycine and glutamate, is one of the precursors of glutathione (White et al., 2003). Cysteine is the rate-limiting substrate of glutathione production, revealing an important role for NAC in replenishing these stores (Sen, 2001). Indeed, NAC administration directly increases production of glutathione (Atkuri et al., 2007). We believe *nnt* mutants are unable to effectively reduce NADP+ to NADPH, leading to fewer stores of reduced glutathione. Thus, NAC was chosen as a means of compensating for this potential decrease in reduction capacity.

**Figure 5:**
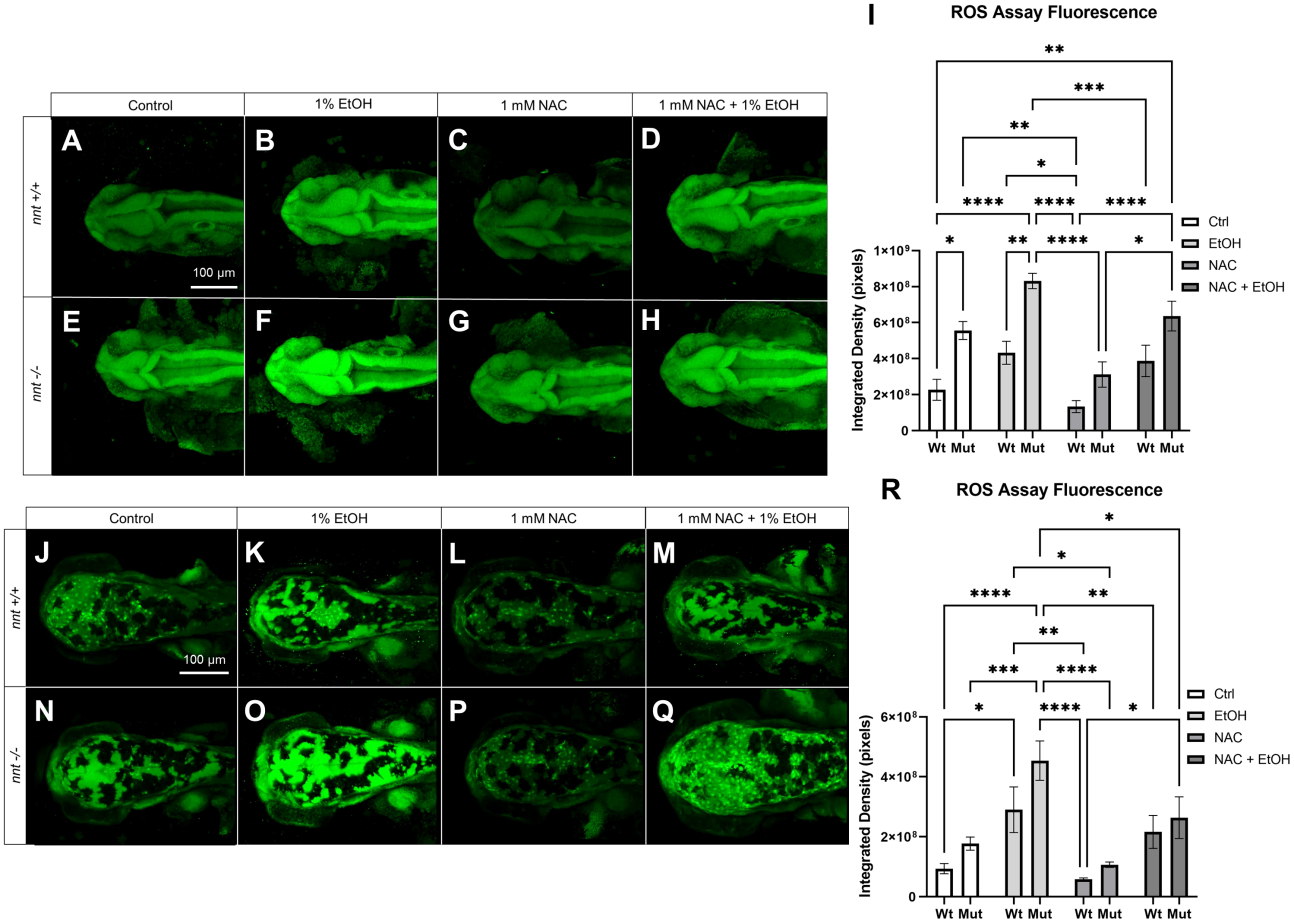
ROS concentration is elevated in *nnt* mutants exposed to ethanol. **(A–H)** CellROX Assay-stained 24 hpf fish that were unexposed, exposed to 1% EtOH from 6–24 hpf, exposed to NAC from 6–24 hpf, or exposed to 1% EtOH +1 mM NAC from 6–24 hpf. All images are dorsal views of the head with anterior to the left. **(A)** Unexposed wildtype embryos have lower basal levels of ROS than **(E)** unexposed mutants (*p* = 0.0175). **(B)** 1% EtOH exposed wildtypes have lower ROS levels than **(F)** EtOH-exposed mutants (*p* = 0.0022). **(C)** 1 mM NAC treated wildtypes were not significantly different from **(G)** NAC treated mutants. **(D)** Wildtypes treated with 1 mM NAC and 1% EtOH were not significantly different from the **(H)** NAC and EtOH-treated mutant, but these mutants had significantly elevated ROS compared to the wildtype control (*p* = 0.0016). **(I)** Graph depicting ROS concentration across all groups in 24 hpf zebrafish (Two-way ANOVA with multiple comparisons, black bars depict mean ± SEM, *n* = 5 per group, *p < 0.05, **p < 0.01, ***p < 0.001, ****p < 0.0001). **(J–Q)** CellROX Assay-stained 48 hpf fish. Dorsal views, anterior to the left. **(J)** Unexposed wildtypes were not significantly different in ROS concentration than **(N)** unexposed mutants. **(K)** 1% EtOH-exposed wildtypes were not significantly different from **(O)** EtOH-exposed mutants. **(L)** Wildtypes treated with 1 mM NAC were not significantly different from **(P)** NAC-treated mutants. **(M)** Wildtypes treated with 1 mM NAC and 1% EtOH were not significantly different from the **(Q)** NAC and EtOH-treated mutant, though these mutants had significantly lower ROS concentration compared to the EtOH-treated mutants (*p* = 0.0337). **(R)** Graph depicting ROS concentration across all groups in 48 hpf zebrafish (Two-way ANOVA with multiple comparisons, black bars depict mean ± SEM, *n* = 5 per group, *p < 0.05, **p < 0.01, ***p < 0.001, ****p < 0.0001). Ctrl: control, EtOH: ethanol, NAC: N-acetyl Cysteine, Mut: mutant, Wt: wildtype.

To assess ROS concentration in *nnt* mutants compared to wildtypes, the CellROX ROS Assay Kit was used to stain 24 hpf and 48 hpf embryos (Figure 5). Embryos were untreated, dosed with 1% ethanol, dosed with 1 mM NAC, or treated with 1 mM NAC + 1% ethanol from 6 to 24 hpf. There was a significant increase in the basal concentration of ROS at 24 hpf in both unexposed and ethanol treated mutants compared to the respective wildtypes (*p* = 0.0175, *p* = 0.0022, respectively) (Figures 5A,B,E,F,I). While there was a decrease in ROS level in the *nnt* mutants treated with NAC + EtOH compared to those treated solely with ethanol, this change did not reach *p* < 0.05 (*p* = 0.3915) (Figures 5F,H,I). We note though that this level of ROS was similar to that in ethanol-exposed wildtypes, which develop no profound defects. When treated with NAC alone however, the mutant has a significantly lower level of ROS than the NAC + EtOH treated mutant (*p* = 0.0201) (Figures 5G–I). At 48 hpf, there were still significantly elevated levels of ROS in ethanol-exposed *nnt* mutants compared to untreated mutants and wildtypes (*p* = 0.0005, *p* < 0.0001) (Figures 5J,N,O,R). There was a significant difference between the ethanol-treated *nnt* mutant and the NAC + EtOH treated mutant (*p* = 0.0337) (Figures 5O,Q,R). Mutants treated solely with NAC were not significantly different from the control mutants, but they had significantly lower levels of ROS compared to the ethanol-treated mutants (*p* = 0.9067, *p* < 0.0001, respectively) (Figures 5N–P,R). Collectively, these data show that ethanol exposure further exacerbates an elevation in ROS levels in *nnt* mutants and that NAC can partially restore the level of ROS.

**Figure 6:**
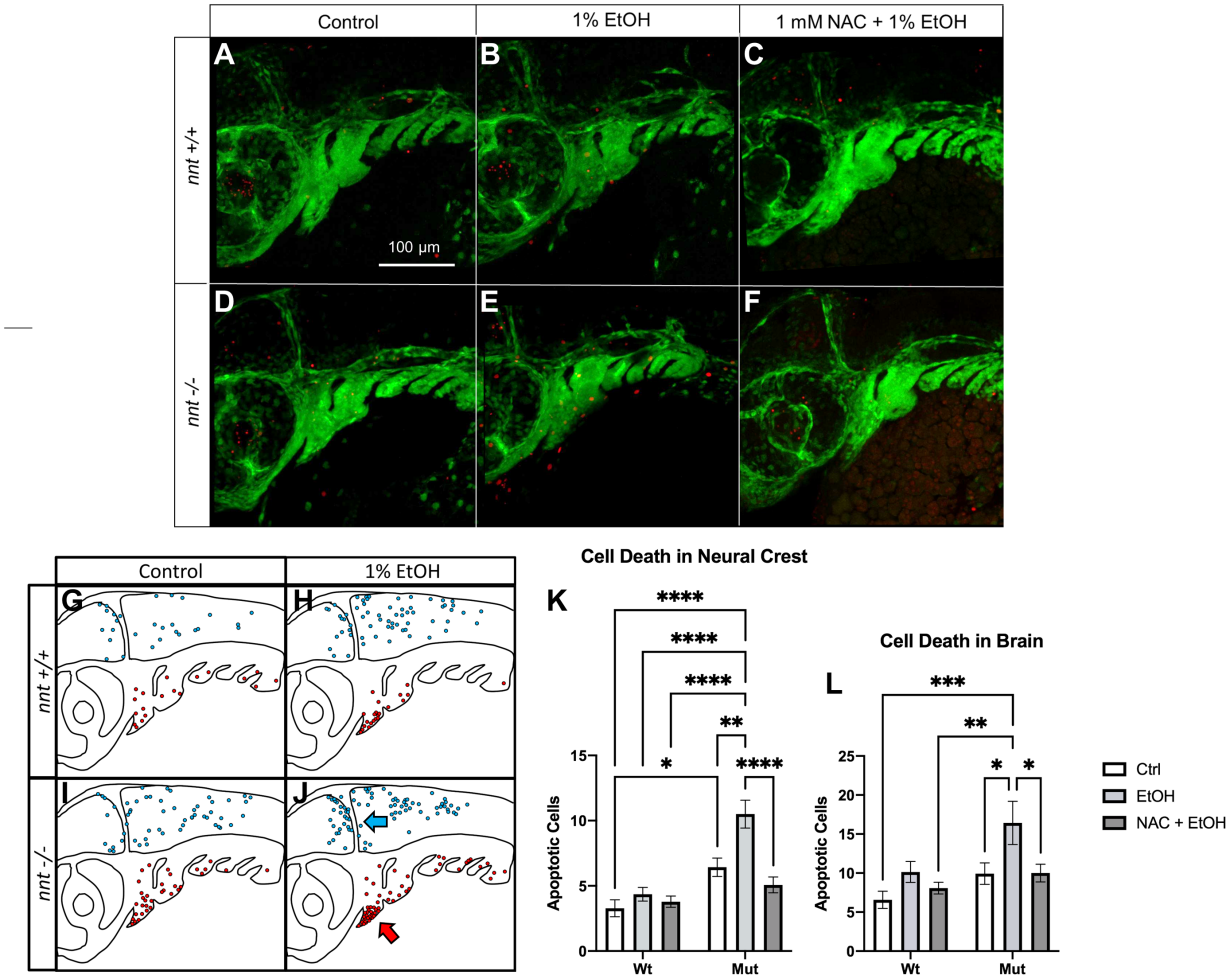
Aberrant ethanol-induced apoptosis in *nnt* mutants is rescued by antioxidant treatment. **(A–F)** 36 hpf *nnt;fli1a:eGFP* (green) embryos stained with TUNEL (red) (*n* = 14 per group). Lateral views, anterior to the left, dorsal up. **(A)** Unexposed wildtypes had significantly lower apoptosis in the pharyngeal arches compared to **(D)** unexposed mutants (*p* = 0.0230). **(B)** 1% ethanol-dosed wildtypes had significantly less apoptosis in the arches and brain compared to **(E)** ethanol-treated mutants (*p* = <0.0001 and *p* = 0.0003). **(C)** 1 mM NAC + 1% EtOH dosed wildtypes were not significantly different from **(F)** NAC + EtOH-treated mutants (*p* = 0.7775), but NAC + EtOH mutants had significantly lower apoptosis in the arches and brain compared to ethanol-dosed mutants (*p* = <0001 and *p* = 0.0491, respectively). **(G–J)** Schematic of the spatial distribution of apoptosis in the brains (blue arrow) and pharyngeal arches (red arrow) of untreated and ethanol-treated mutants and wildtypes (*n* = 5 per group). **(G)** The wildtype control had fewer apoptotic cells that were more widely distributed across the brain and arches compared to the **(H,I)** unexposed mutants and ethanol-treated wildtypes that had higher levels of apoptosis in both regions. **(J)** Ethanol-treated mutants had more apoptosis than all other groups and had cell death localized to the ventral portion of the arches and clustered around the midbrain-hindbrain boundary. **(K)** Graph depicting apoptotic cells in the neural crest across all groups (Two-way ANOVA with multiple comparisons, black bars depict mean ± SEM, *n* = 14 per group, *p < 0.05, **p < 0.01, ****p < 0.0001). **(L)** Graph depicting apoptotic cells in the brain across all groups (Two-way ANOVA with multiple comparisons, black bars depict mean ± SEM, *n* = 14 per group, *p < 0.05, **p < 0.01, ***p < 0.001). Ctrl: control, EtOH: ethanol, NAC: N-acetyl Cysteine, Mut: mutant, Wt: wildtype.

Elevated ROS can lead to apoptosis. We performed TUNEL in a *nnt;fli1a:eGFP* transgenic line to quantify cell death (Figure 6). TUNEL was performed on 36 hpf embryos that were untreated or dosed with 1% ethanol from 6 to 24 hpf. We counted apoptotic neural crest cells (GFP positive) and cells within the brain (evident in the PMT channel). Interestingly, unexposed *nnt* mutants had significantly increased basal level of cell death in the pharyngeal arches relative to the unexposed *nnt* wildtypes (*p* = 0.0230) (Figures 6A,D,K). When treated with ethanol, *nnt* mutants experience significantly elevated levels of apoptosis in the arches relative to the wildtype ethanol group (*p* < 0.0001) (Figures 6B,E,K). The most significant elevation in apoptosis in the arches occurs largely in arches 1 and 2, consistent with cartilages derived from these arches being most sensitive to disruption (Supplementary Figure S2). Cell death also appears localized to the ventral portions of arches 1 and 2 where progenitor cells for Meckel’s cartilage and the ceratohyal are localized (Figure 6J). In the brain, there is not a significant difference between the unexposed *nnt* mutant and wildtype (*p* = 0.6465) (Figures 5A,D,L). With the addition of ethanol, there is a significant increase in apoptosis in the brains of *nnt* mutants relative to unexposed *nnt* wildtypes (*p* = 0.0003) (Figures 6A,E,L). As in the neural crest, there appears to be a spatially restricted distribution of cell death, with an abundance of cell death that appears to be localized around the midbrain-hindbrain boundary (MHB) (Figure 6J).

We hypothesize that if elevated ROS is causing this increase in apoptosis, antioxidant administration may then be able to rescue this defect. To test this prediction, we performed TUNEL on 36 hpf embryos from the *nnt;fli1a;eGFP* transgenic line that were treated with 1 mM NAC + 1% ethanol from 6–24 hpf (Figures 6C,F,K,L). There was a significant decrease in the apoptosis of the arches in ethanol + NAC-treated *nnt* mutants compared to those treated with ethanol alone (*p* < 0.0001) (Figures 6E,F,K). In the brain, there is also a significant decrease of cell death in ethanol + NAC-treated *nnt* mutants compared to those treated with ethanol (*p* = 0.0491) (Figures 6E,F,L). We conclude that nnt function protects sensitive cell types, such as neural crest cells and neurons, from ROS-induced apoptosis.

**Figure 7:**
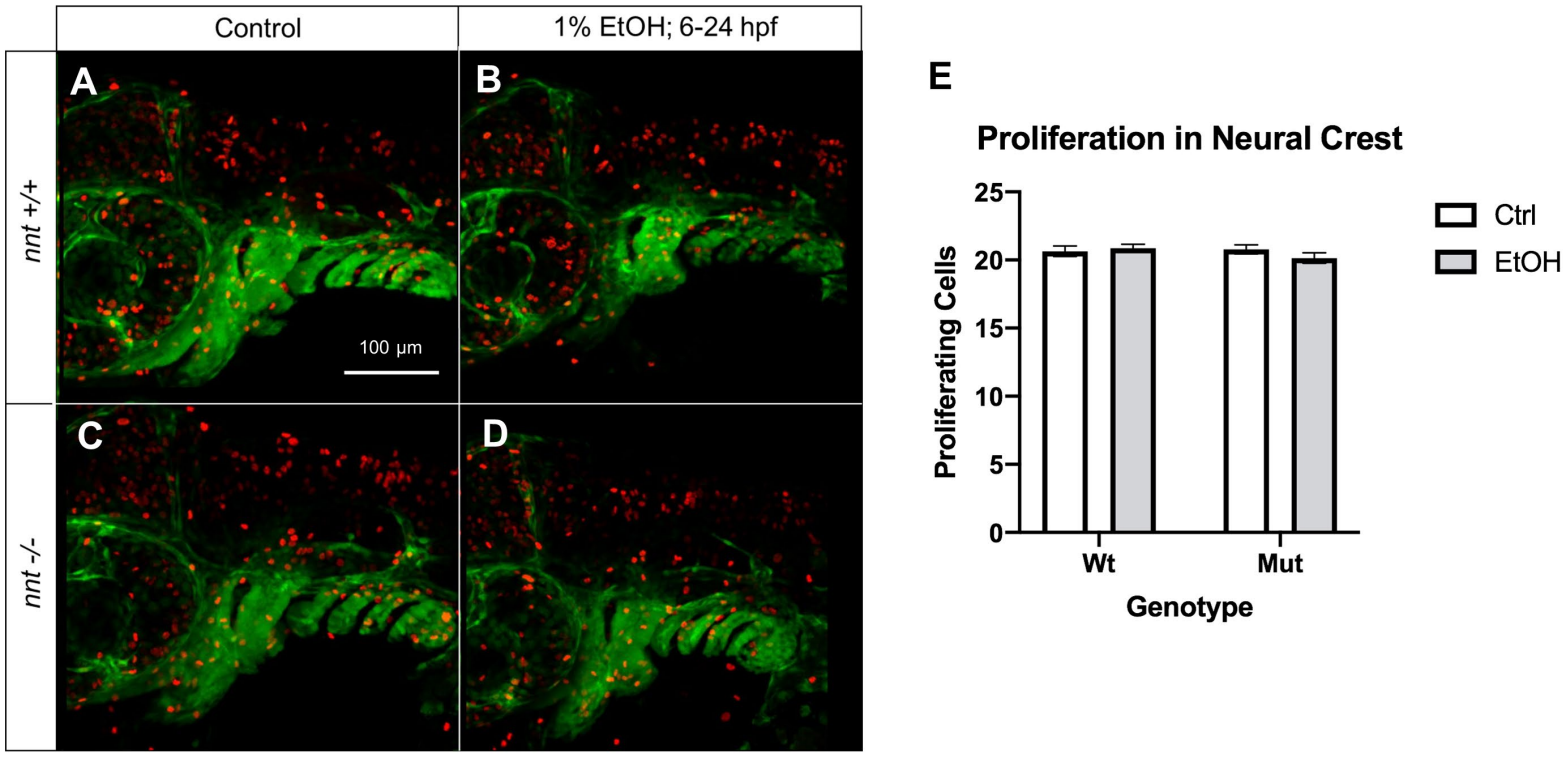
Proliferation is not significantly altered in neural crest of *nnt* mutants. **(A–D)** 36 hpf *nnt;fli1a:eGFP* (green) embryos stained with pHH3 (red). Lateral views, anterior to the left, dorsal up. **(A)** Unexposed wildtypes and **(C)** mutants have similar levels of proliferating cells compared to **(B)** ethanol-treated wildtypes and **(D)** mutants. **(E)** Graph depicting proliferating cells in the neural crest (Twoway ANOVA with multiple comparisons, black bars depict mean ± SEM, *n* = 14 per group). Ctrl: control, EtOH: ethanol, Mut: mutant, Wt: wildtype.

An additional cellular mechanism for the observed craniofacial defects could be reduced neural crest cell proliferation. Thus, we also analyzed proliferation in these embryos using the proliferation marker pHH3 (Figure 7). Embryos were untreated or dosed with 1% ethanol from 6–24 hpf and fixed for staining at 36 hpf. We found no differences across any of the treatment or genotype groups. Collectively, these results demonstrate that *nnt* mutants are sensitized to ethanol-induced cell death.

**Figure 8:**
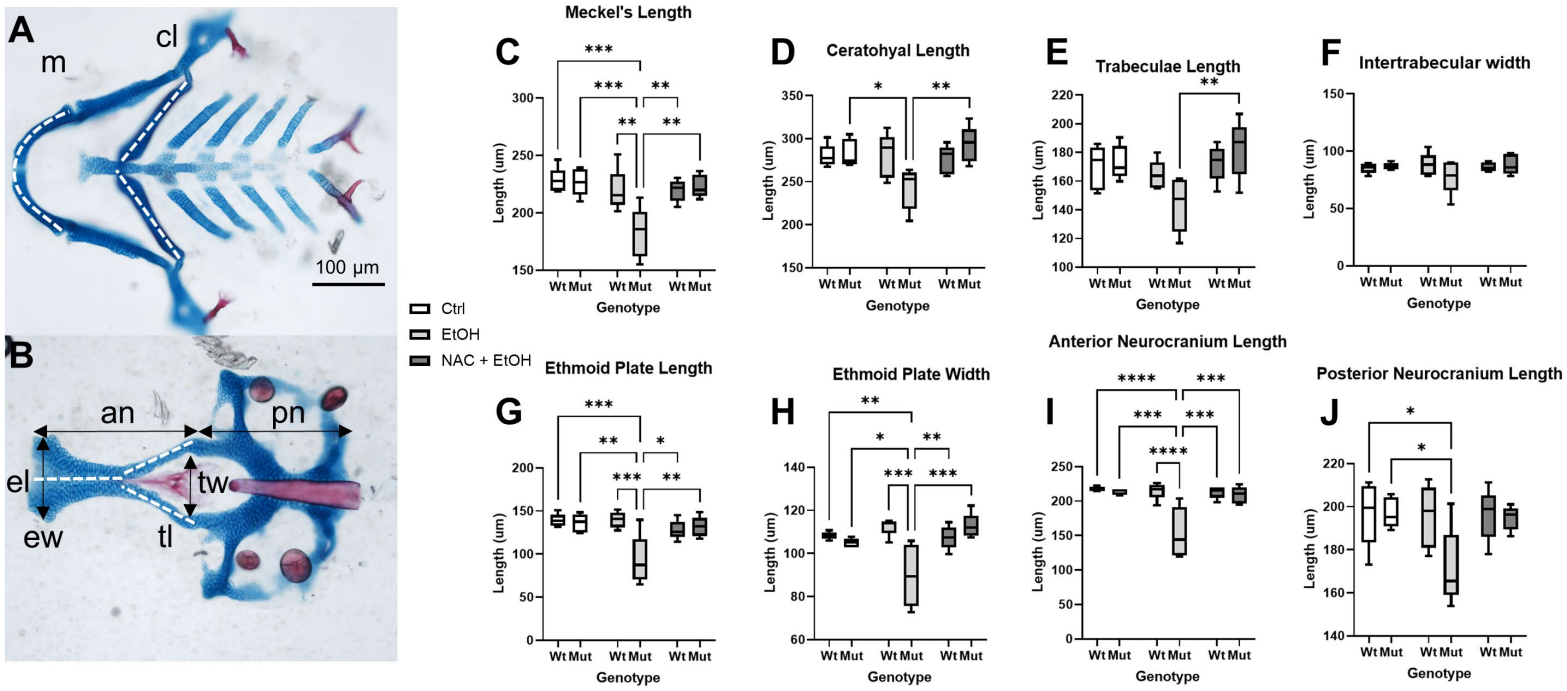
Ethanol-induced craniofacial abnormalities are rescued by concurrent NAC dosage. **(A,B)** Flatmounts of 5 dpf wildtype craniofacial skeleton displaying the linear measurements used with anterior to the left. **(A)** Viscerocranium and **(B)** neurocranium. The cartilaginous elements that were measured: m: Meckel’s length, cl: ceratohyal length, tl: Trabeculae length, tw: Intertrabecular width, el: Ethmoid plate length, ew: Ethmoid plate width, an: Anterior neurocranium length, pn: Posterior neurocranium length. **(C–J)** Embryo were unexposed, exposed to 1% EtOH from 6–24 hpf, or exposed to 1% EtOH +1 mM NAC from 6–24 hpf. Fish were then grown to 5 dpf for morphometric analyses. Graphs depict craniofacial measurements across each cartilaginous element in all groups (Two-way ANOVA with multiple comparisons, black bars depict range, *n* = 5 per group, *p < 0.05, **p < 0.01, ***p < 0.001, ****p < 0.0001). In nearly every instance in which ethanol caused a significant reduction in length or width in *nnt* mutants, the co-exposure with NAC restored the size back to that of wildtype **(C,D,G-I)**. The only exception being posterior neurocranium length, where the co-exposure is not significantly different from wildtype or ethanol-exposed *nnt* mutants. Ctrl: control, EtOH: ethanol, NAC: N-acetyl Cysteine, Mut: mutant, Wt: wildtype.

Our model further predicts that this ROS-induced apoptosis causes craniofacial defects in *nnt* mutants. Therefore, we quantified cartilaginous defects in the face to determine the extent to which antioxidant treatment ameliorates the craniofacial defects in ethanol-exposed *nnt* mutants. We took linear measurements of the neural crest-derived Meckel’s cartilage, ceratohyal, trabeculae, ethmoid plate and anterior neurocranium (Figure 8). We also measured the mesoderm-derived posterior neurocranium and quantified inter-trabeculae width as a midface measure. None of these measures varied between untreated wildtype, untreated mutant, ethanol-treated wildtype fish and ethanol + NAC-treated wildtype fish. No differences were found across groups for inter-trabeculae width. For all other measures except trabeculae length, ethanol-exposure significantly reduced the size of the cartilage element, although we note the strong trend in trabeculae length. The reduction in overall head size (noted in Figure 2) would be explained by the reduction of both neural crest-derived and mesodermally-derived portions of the skull. There was no significant difference in body length among any of the groups (Supplementary Figure S3). For all of these measures, NAC treatment restored the size of the skeletal element relative to control. For a complete list of comparisons, see Supplementary Table S1. These results demonstrate that antioxidant treatment protects *nnt* mutants from ethanol teratogenesis.

## Discussion

Our findings have demonstrated that loss of Nnt function in and of itself leads to increased susceptibility to alcohol teratogenesis. In addition, exposed mutants display severe craniofacial defects at normally subteratogenic doses of alcohol. We have shown that *nnt* mutants have greatly elevated levels of ROS following ethanol exposure. This elevated ROS leads to elevated levels of apoptosis in sensitive cell types and causes the observed craniofacial defects. Our findings have broad implications not only for researchers using C57 mice, but for humans as well, due to high level of conservation of *nnt*. Factors that elevate ROS, environmentally or genetically, could interact and lead to defects. Any phenotype that could be modified by ROS could be affected in a C57/B6J background and should be considered in future research.

### Role of Nnt in oxidative stress

The primary function of Nnt is in reducing NADP+ to NADPH. NADPH is an important cofactor in many metabolic pathways such as cellular respiration and ATP production. This reducing agent also plays a critical role in detoxifying ROS (Zamzami et al., 1995). NADPH reduces the oxidized form of glutathione (GSSG) into a reduced state (GSH) such that glutathione is now able to directly sequester and reduce ROS.

We hypothesize that in an embryo where the initial reduction of NADP+ to NADPH is compromised, inadequate stores of GSH will be present to reduce ROS. The administration of ethanol is known to increase ROS (Henderson et al., 1995, 1999; Chu et al., 2007; Brocardo et al., 2011; Yan and Zhao, 2020). Thus, in a *nnt* mutant background, this ROS would lead to elevated levels of oxidative stress and subsequent apoptosis. Based on our ROS assay, we do observe significantly higher basal levels of ROS in mutants at 24 hpf. With the addition of ethanol, mutants have significantly elevated ROS concentration. The elevation at 48 hpf, 24 h after the embryos were last exposed to ethanol was surprising, but demonstrates that the ROS induced by ethanol is having a lasting effect. The concurrent dosage of NAC + EtOH in mutants was found to significantly decrease ROS concentration at 48 hpf. Dosage of NAC alone did not significantly lower ROS concentration relative to the control, however there does appear to be a trend toward reduction. In the 24 hpf embryos however, the NAC treated mutants have significantly lower levels of ROS compared to the NAC + EtOH treated mutants (*p* = 0.0201). NAC appears to reduce the basal level of ROS, likely due to its ability to directly upregulate glutathione. To better define the mechanism of *nnt* in mitigating oxidative stress, direct measurements of the GSH/GSSG ratio and levels of NADP+/NADPH could be performed.

We utilized NAC to mitigate the deleterious effects of this increased oxidative stress. Studies on diet during pregnancy suggest antioxidants such as vitamin E, beta-carotene, selenium, choline and folic acid protect against ethanol-induced birth defects (Mitchell et al., 1999; Ojeda et al., 2009; May et al., 2014b). Though NAC use in pregnant mothers with alcohol-exposed fetuses has not been well-characterized, human data suggests that NAC during pregnancy has neuroprotective effects (Wiest et al., 2014). The existing literature and our experimentation provide evidence for the amelioration of ethanol-induced birth defects via antioxidant administration.

### Cellular mechanism and craniofacial defects

Unexposed *nnt* mutants experienced higher levels of apoptosis in the pharyngeal arches relative to their wildtype counterparts. This is a surprising phenotype given that they do not appear to have an aberrant craniofacial phenotype in the absence of ethanol. We have previously shown that ethanol increases cell death in wildtype zebrafish (McCarthy et al., 2013). However, this level of apoptosis is not elevated to the point of causing craniofacial defects. We believe that there may be a threshold effect where there is a certain level of cell death that needs to occur for craniofacial development to be perturbed. The localization of the cell death at the most ventral portions of arches 1 and 2 is consistent with the craniofacial phenotype as these cells give rise to the most anterior craniofacial elements which are disrupted in exposed mutants (Eberhart et al., 2006). Apoptosis of the neural crest is thought to contribute to the craniofacial phenotype of FASD as shown by various animal studies (Cartwright and Smith, 1995; Dunty et al., 2001). The morphology of these fish is consistent with FASD-associated malformations, such as an underdeveloped jaw and microcephaly (Jones et al., 1973; Jackson and Hussain, 1990).

In addition to recapitulating the craniofacial phenotype associated with FASD, ethanol-exposed mutants also exhibited apoptosis in areas of the brain affected by prenatal ethanol exposure. When mutants were exposed to ethanol, clustering of apoptotic cells occurred, particularly around the MHB. The MHB gives rise to the cerebellum, a structure responsible for motor function. Children with FASD often experience cerebellar defects (West, 1993; Swayze et al., 1997; Norman et al., 2009). Prenatally exposed children often experience a decrease in volume of both the cerebellum and the vermis (Autti-Rämö et al., 2002). An interesting future experiment could be to track this cell death in the developed cerebellum to determine if specific subpopulations of cells are lost.

### Role of Nicotinamide nucleotide transhydrogenase across vertebrate species

The NNT transhydrogenase is highly conserved across species, with zebrafish and human having 82.23% identity (Cunningham et al., 2022). The high conservation of this gene is likely due to the critical function of NNT. Loss of function of this gene in humans is predicted to be strongly selected against as the loss-of-function observed/expected upper bound fraction (LOEUF) is quite low at 0.311 (Karczewski et al., 2020). Thus, the fact that loss of Nnt is tolerated in laboratory-raised mouse and zebrafish is somewhat surprising. The critical function of Nnt is only revealed when mutants are environmentally challenged. Thus, as we have found with *platelet-derived growth factor receptor alpha* (*pdgfra*) mutants, environmental exposures can reveal requirements for gene function not demonstrated in neutral laboratory conditions (McCarthy et al., 2013).

While associations between FASD and *NNT* in humans have not been characterized, *NNT* dysfunction in humans is associated with heart failure. In human cardiac tissue derived from donors with chronic severe heart failure, NNT activity was 18% lower than donors who did not experience heart failure (Sheeran et al., 2010). This change was accompanied by lower GSH/GSSG ratio, lower NADPH levels, and higher levels of oxidized glutathione. This may be due to the heart’s inability to maintain proper metabolic function and antioxidant defense.

Mutations in *NNT* are also associated with glucocorticoid deficiency 1 (GCCD1) (Meimaridou et al., 2012; Weinberg-Shukron et al., 2015). GCCD1 is characterized by inability of the adrenal cortex to respond to adrenocorticotropin hormone and produce cortisol. SNPs in *NNT* were found in 15 kindreds with GCCD1 (Meimaridou et al., 2012). In a human adrenocortical cell line, loss of *NNT* resulted in increased superoxide concentration as well as lower GSH/GSSG ratios, suggesting that inability to properly regulate oxidative stress may contribute to adrenal dysfunction.

C57BL/6J mice also experience redox abnormalities, with 6J mice exhibiting increase superoxide production (Leskov et al., 2017). 6J mice also have increased mitochondrial membrane permeability, spontaneous NADPH oxidation, and lower GSH/GSSG ratios compared to a 6J strain with wildtype *Nnt* (Huang et al., 2006). Collectively, these studies demonstrate the importance of Nnt in modulating ROS and subsequent cellular functions.

Our research has demonstrated that loss of *nnt* sensitizes embryos to ethanol-induced defects. The importance of this gene across multiple species strongly suggests that NNT function may be involved in FASD. Our findings demonstrate that nnt is a critical modulator of ROS levels following ethanol exposure, to prevent apoptosis of sensitive cell types. Our findings provide insight into the genesis of FASD and will help to guide treatments to prevent FASD.

## Data availability statement

The raw data supporting the conclusions of this article will be made available by the authors, without undue reservation.

## Ethics statement

The animal study was reviewed and approved by the University of Texas at Austin Institutional Animal Care and Use Committee.

## Author contributions

JKE and RM conceived and designed the study and wrote the manuscript. JKE generated the CRISPR mutants. RM carried out all subsequent experiments and analyses. All authors contributed to the article and approved the submitted version.

## Funding

This work was funded by NIH/NIAAA R01AA023426, NIH/NIDCR R35DE029086, and NIH/NIAAA 5U01AA021651 to JKE, and NIH/NIAAA 2T32AA007471–36 to RM.

## Conflict of interest

The authors declare that the research was conducted in the absence of any commercial or financial relationships that could be construed as a potential conflict of interest.

## Publisher’s note

All claims expressed in this article are solely those of the authors and do not necessarily represent those of their affiliated organizations, or those of the publisher, the editors and the reviewers. Any product that may be evaluated in this article, or claim that may be made by its manufacturer, is not guaranteed or endorsed by the publisher.

## Footnotes

^1^ http://zifit.partners.org/ZiFiT/
