# Supplementary material for: Loss of Nicotinamide nucleotide transhydrogenase sensitizes embryos to ethanol-induced neural crest and neural apoptosis via generation of reactive oxygen species
Source: Front Neurosci. 2023 Jun 9;17:1154621. doi: 10.3389/fnins.2023.1154621 (PMC10289183; doi:10.3389/fnins.2023.1154621)
Supplement: Supplementary file 2 [file Data_Sheet_1.PDF]

Supplementary Material

Loss of *nicotinamide nucleotide transhydrogenase* sensitizes embryos to ethanol-induced neural crest and neural apoptosis via generation of reactive oxygen species

Rayna Mazumdar*, Johann Eberhart

*** Correspondence:** Corresponding Author: rayna.mazumdar@utexas.edu

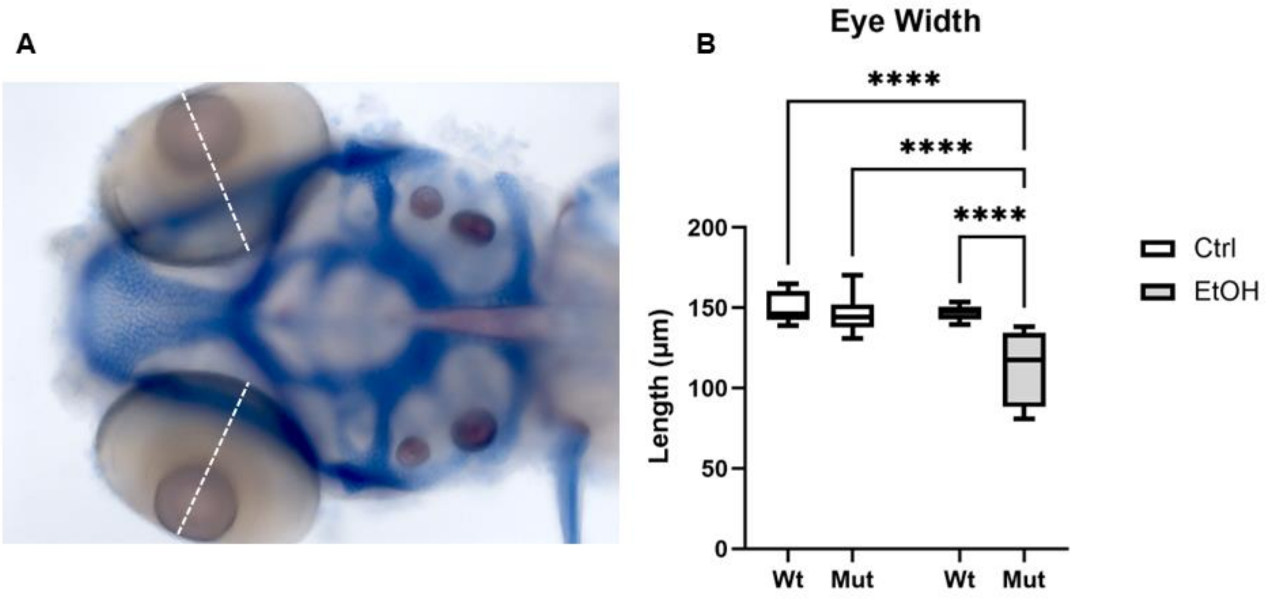

Supplemental 1. Ethanol exposure reduces eye width of *nnt* mutants

(A) Whole mount of 5 dpf wildtype embryo depicting eye width measures (dashed lines). (B) Graph depicting eye width measures (Two-way ANOVA with multiple comparisons, black bars depict range, n = 5 per group, **** = $p < .0001$).

.

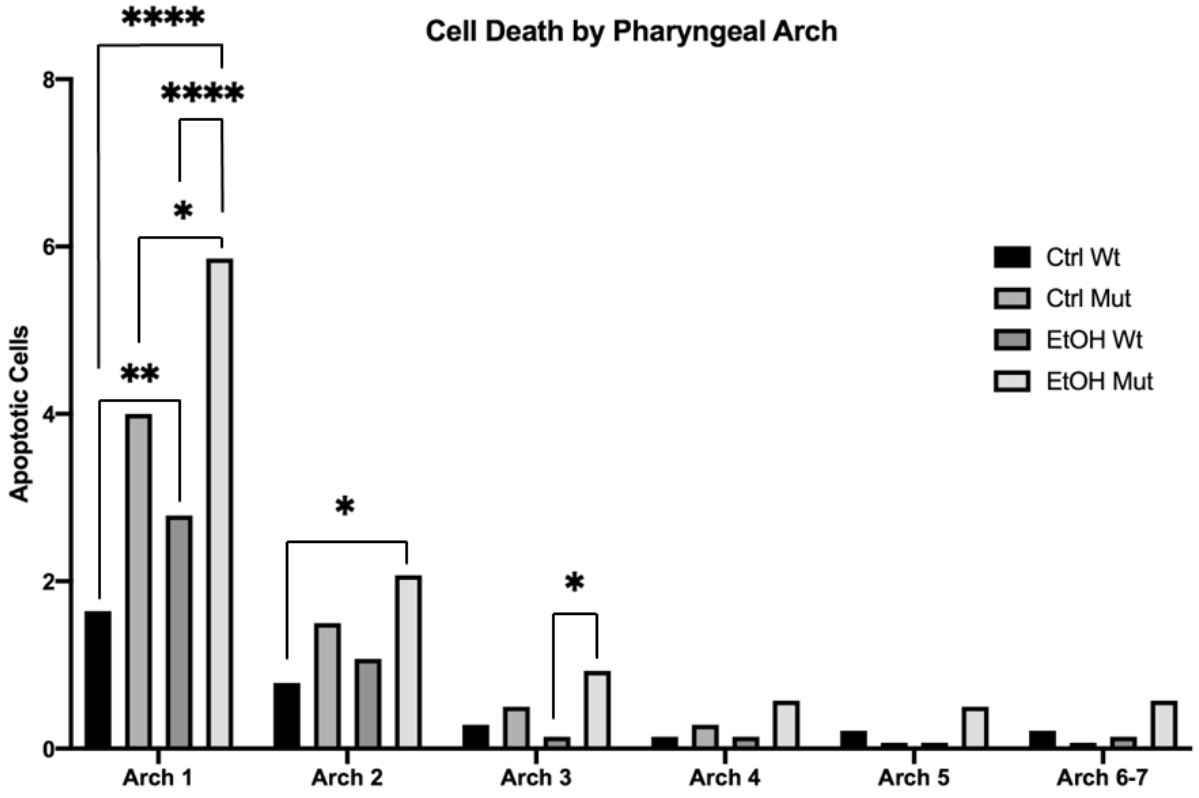

Supplemental 2. Cell death is elevated in the first and second pharyngeal arches of *nnt* mutants

Graph depicting the amount of cell death per arch across treatment and genotype (Two-way ANOVA with multiple comparisons, n = 5 per group, * = $p < .05$, ** = $p < .01$, **** = $p < .0001$). Ctrl: control, EtOH: ethanol, Mut: mutant, Wt: wildtype

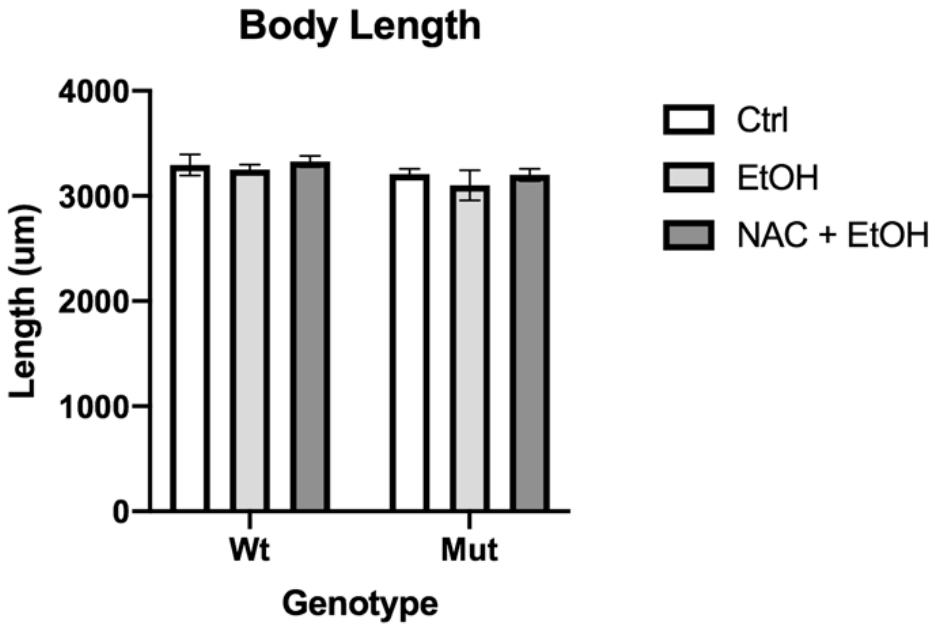

Supplemental 3. Body length is not significantly altered by genotype or treatment

Graph depicting body length measures across all groups (Two-way ANOVA with multiple comparisons, black bars depict mean ± SEM, n = 5 per group)

	Meckel's Length	Ceratohyal Length	Intertrabecular Width	Trabeculae Length	Ethmoid Plate Length	Ethmoid Plate Width	Anterior Neurocranium Length	Posterior Neurocranium Length
Wt:Ctrl vs. Wt:EtOH	0.9321	>0.9999	0.9963	0.991	>0.9999	0.9277	>0.9999	>0.9999
Wt:Ctrl vs. Wt:NAC + EtOH	0.935	0.9994	>0.9999	0.9996	0.8588	>0.9999	0.9985	>0.9999
Wt:Ctrl vs. Mut:Ctrl	>0.9999	>0.9999	0.9995	0.9991	0.9995	0.9713	0.9977	>0.9999
Wt:Ctrl vs. Mut:EtOH	**0.0006**	0.0636	0.7742	0.1146	**0.0007**	**0.0038**	**<0.0001**	**0.0429**
Wt:Ctrl vs. Mut:NAC + EtOH	0.9942	0.8979	0.9925	0.7681	0.9687	0.9123	0.9656	0.9997
Wt:EtOH vs. Wt:NAC + EtOH	>0.9999	0.9986	0.9995	0.9463	0.8173	0.8613	>0.9999	>0.9999
Wt:EtOH vs. Mut:Ctrl	0.9656	>0.9999	>0.9999	0.9307	0.9983	0.5346	>0.9999	>0.9999
Wt:EtOH vs. Mut:EtOH	**0.006**	0.0562	0.4841	0.3194	**0.0005**	**0.0004**	**<0.0001**	0.0652
Wt:EtOH vs. Mut:NAC + EtOH	0.9984	0.9182	>0.9999	0.419	0.9507	>0.9999	0.9918	>0.9999
Wt:NAC + EtOH vs. Mut:Ctrl	0.9674	0.993	>0.9999	>0.9999	0.9608	0.9914	>0.9999	>0.9999
Wt:NAC + EtOH vs. Mut:EtOH	**0.0059**	0.1249	0.6772	0.0624	**0.0107**	**0.0059**	**0.0002**	0.0521
Wt:NAC + EtOH vs. Mut:NAC + EtOH	0.9986	0.7387	0.9986	0.9043	0.999	0.8394	0.9986	>0.9999
Mut:Ctrl vs. Mut:EtOH	**0.0009**	**0.0397**	0.5897	0.0551	**0.0015**	**0.023**	**0.0002**	**0.0422**
Mut:Ctrl vs. Mut:NAC + EtOH	0.9987	0.96	0.9998	0.9235	0.9972	0.505	0.9991	0.9996
Mut:EtOH vs. Mut:NAC + EtOH	**0.0022**	**0.0059**	0.4384	**0.006**	**0.0045**	**0.0003**	**0.0004**	0.0796

Table S1. Statistical comparisons of facial measurements

P-value for each comparison across treatment and genotype for all facial measurements. Statistically significant values are bolded (Two-way ANOVA with multiple comparisons, n = 5 per group).
